# Bilateral Inverted Mesiodens: A Rare Case Evaluated by Cone-Beam Computed Tomography

**DOI:** 10.7759/cureus.26629

**Published:** 2022-07-07

**Authors:** Karthik Rajaram Mohan, Ravikumar Pethagounder Thangavelu, Saramma Mathew Fenn

**Affiliations:** 1 Oral Medicine, Vinayaka Mission's Sankarachariyar Dental College, Vinayaka Mission's Research Foundation, Salem, IND; 2 Oral Medicine and Radiology, Vinayaka Mission's Sankarachariyar Dental College, Vinayaka Mission's Research Foundation, Salem, IND

**Keywords:** nasal cavity, supernumerary teeth, mesiodens, anterior nasal spine, conical

## Abstract

Mesiodens is the most commonly occurring supernumerary tooth between the two maxillary central incisors. Mesiodens can be inverted, impacted, or placed buccally or palatally between the two maxillary central incisors. It mostly occurs unilaterally and rarely occurs bilaterally. We describe a rare occurrence of such a bilateral inverted mesiodens extended near the anterior nasal spine, evaluated by cone-beam computed tomography.

## Introduction

Supernumerary teeth are teeth that develop in addition to the normal complement due to excess dental lamina in the jaws [1]. The tooth that develops from such dental lamina may be morphologically normal or abnormal [2]. When such supernumerary teeth have a normal morphological appearance of teeth, they are called supplementary teeth [2]. When they have an abnormal morphological appearance, they are termed mesiodens if they occur between the two maxillary central incisors, peridens if they occur near the premolar region, and distodens if they occur near the molar region [3,4].

## Case presentation

**Figure 1:**
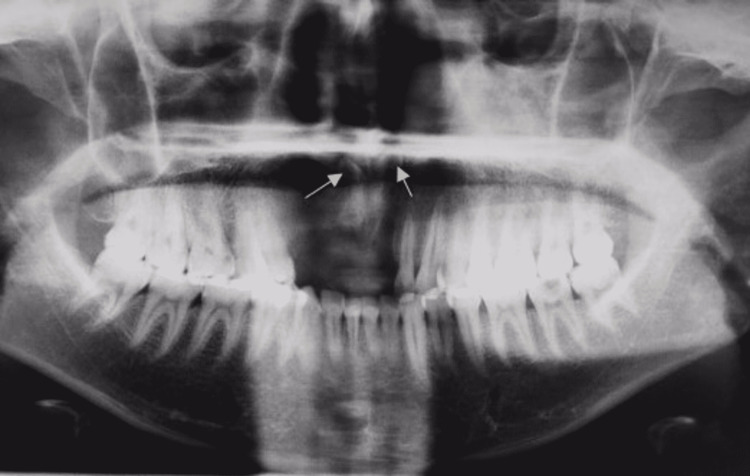
Digital orthopantomography revealed two inverted radiopaque structures extending near the floor of the nasal cavity

**Figure 2:**
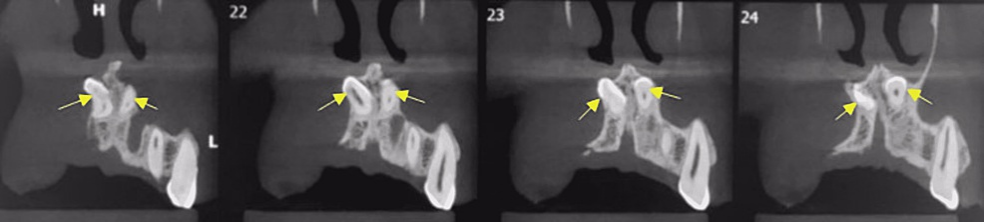
Serial transsagittal sections of cone-beam computed tomography showing bilateral inverted mesiodens

**Figure 3:**
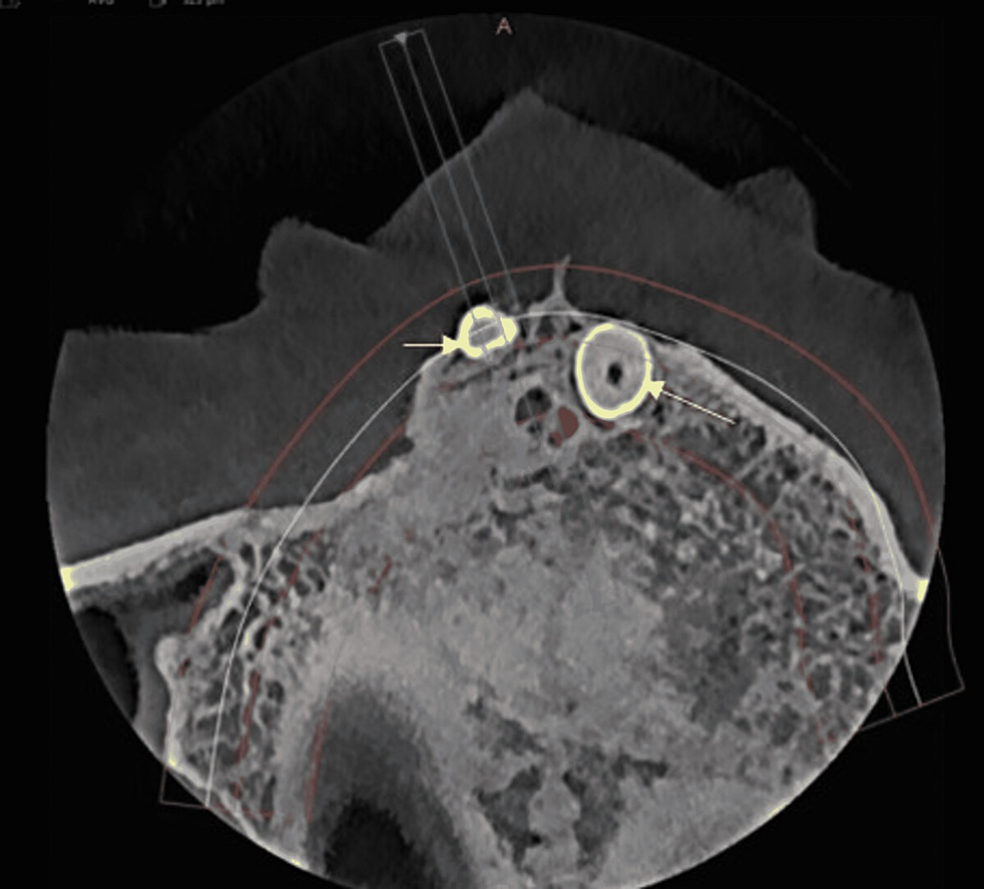
Axial section cone-beam computed tomography revealed bilateral tooth-like radiopacities with a central root canal

**Figure 4:**
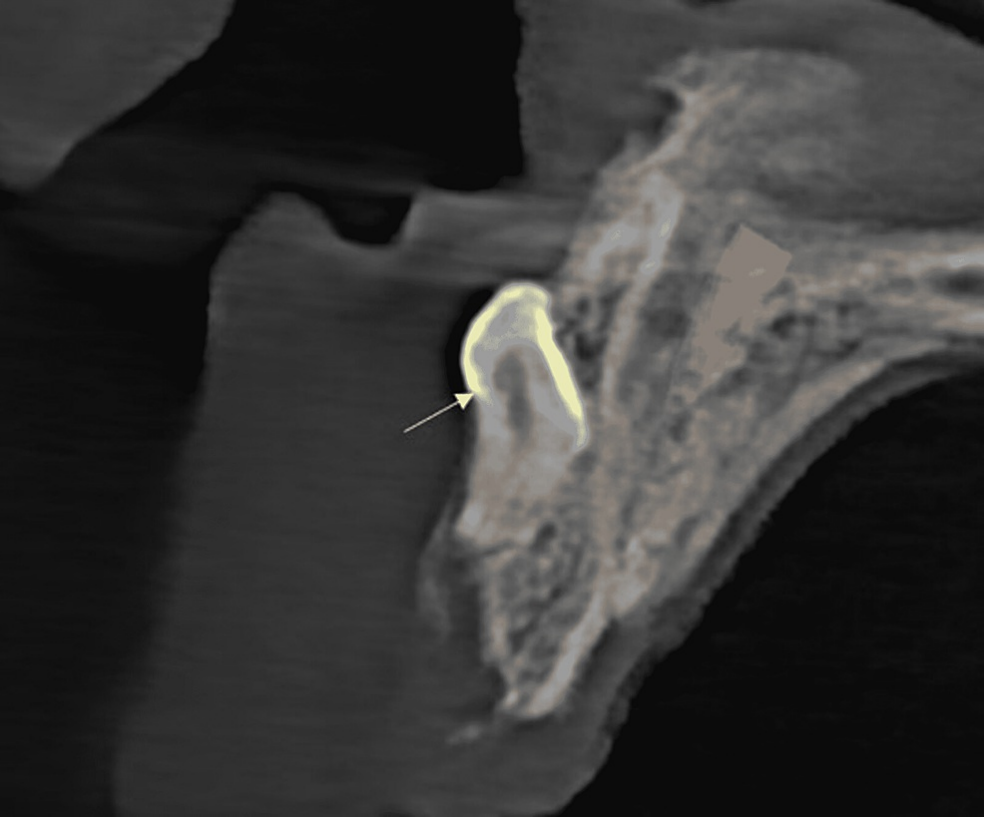
One mesiodens was inverted and attached to the anterior maxillary labial cortical plate

**Figure 5:**
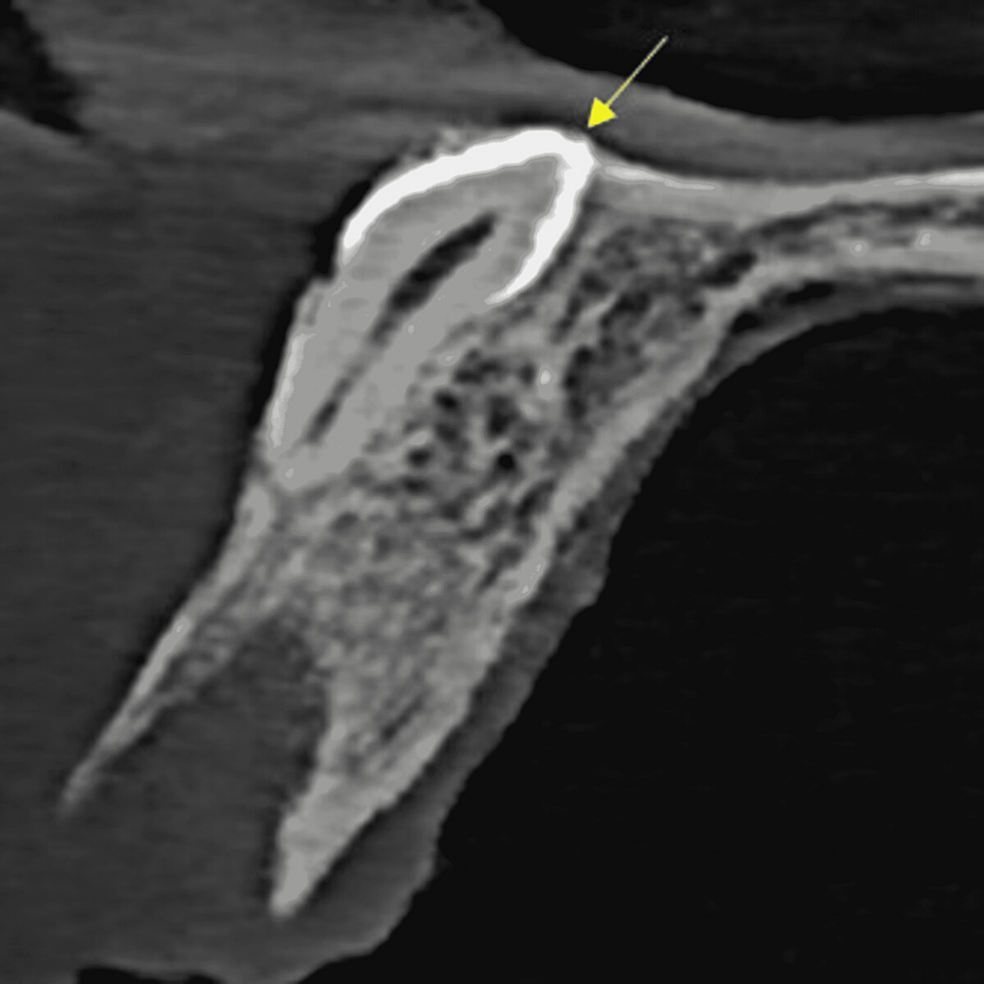
The second inverted mesiodens was extended into the floor of the nasal cavity

**Figure 6:**
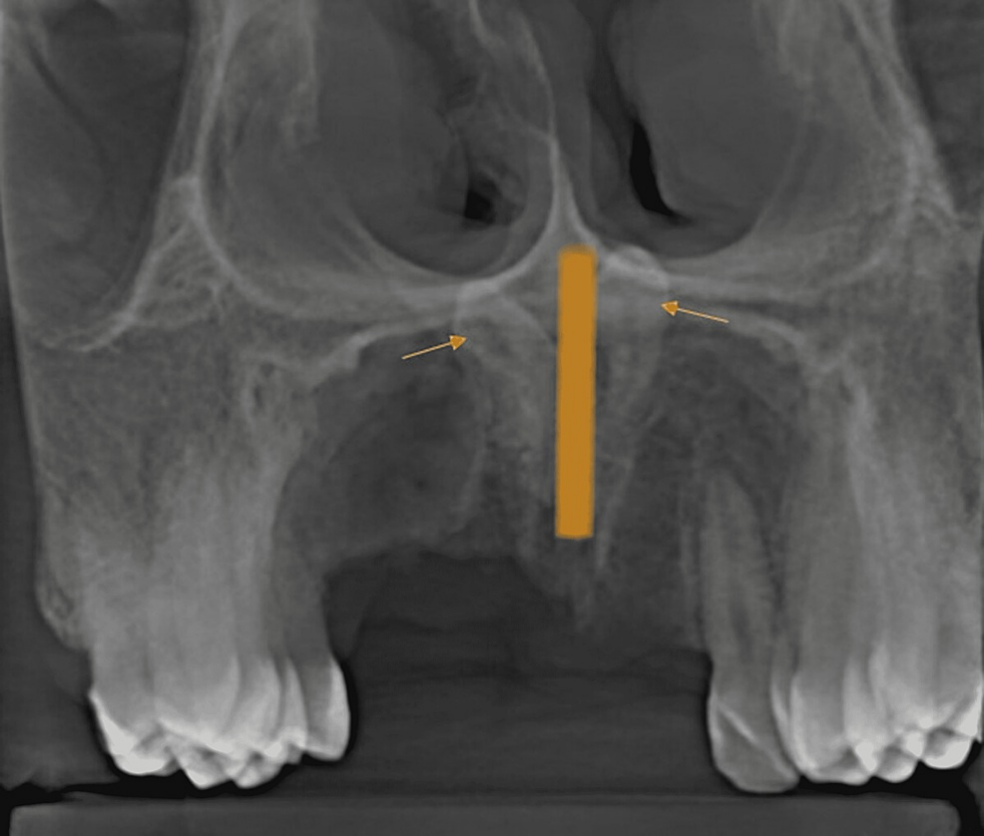
Maximum intensity projection cone-beam computed tomography image with nasopalatine nerve canal tracing done

**Figure 7:**
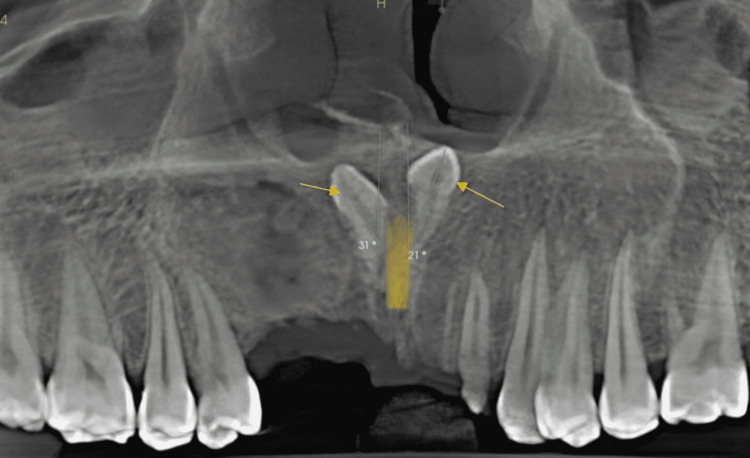
Cone-beam computed tomography with two inverted mesiodens at an angle of 31 and 21 degrees to the nasopalatine nerve canal

**Figure 8:**
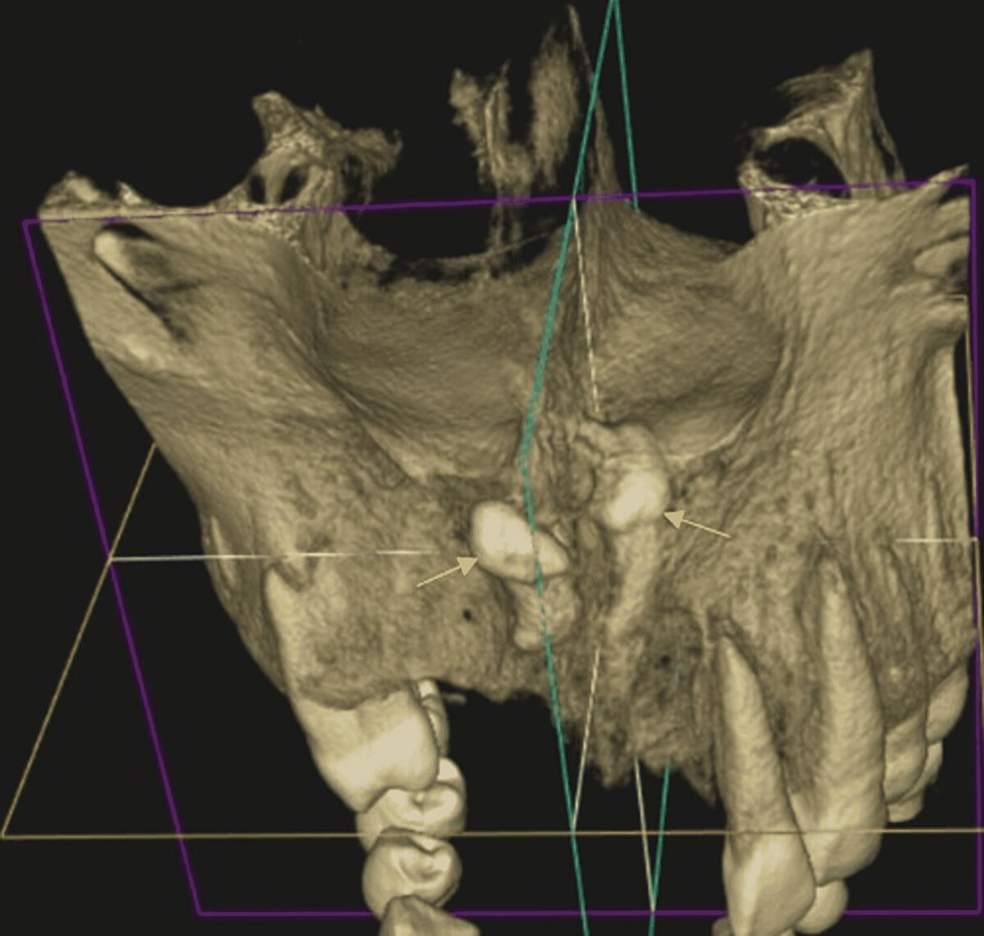
Three-dimensionally reconstructed cone-beam computed tomography image revealed inverted impacted mesiodens

A 21-year-old male reported a chief complaint of missing upper front teeth. A digital orthopantomographic image revealed two inverted radiopaque tooth-like structures near the nasal cavity floor to the anterior nasal spine (Figure 1). The mesiodens teeth were further evaluated by cone-beam computed tomography (CBCT) in serial transcoronal sections (Figure 2). Serial transsagittal images revealed inverted two radiopaque tooth-like structures extending near the floor of the nasal cavity. Axial CBCT revealed two radiopacities with a central radiolucent structure indicating a root canal (Figure 3). Sagittal CBCT revealed one mesiodens attached to the maxillary labial cortical plate (Figure 4). The other mesiodens was also inverted and extended into the floor of the nasal cavity (Figure 5). The maximum intensity projection image of CBCT with nasopalatine canal tracing was depicted (Figure 6). The two mesiodens were at an angulation of 31 and 21 degrees to the nasopalatine canal (Figure 7). A three-dimensionally reconstructed CBCT revealed bilateral inverted mesiodens (Figure 8).

## Discussion

Several theories have been proposed for the genesis of mesiodens. Atavism (phylogenetic reversion) theory states the ancient relic ancestors had three central incisors [5]. Dichotomy theory, in which a tooth bud is split into two separate teeth, usually occurs from complete gemination in the anterior maxilla region [5]. Palatal offshoots or hyperreactivity of active dental lamina are induced to develop into an extra tooth bud, which results in a supernumerary tooth developing into another extra supernumerary tooth [5].

Genetics are also thought to play a vital role in the development of mesiodens since such mesiodens have been diagnosed in siblings, twins, and sequential generations of a single family [5]. Autosomal dominant inheritance with sex-linked patterns with incomplete penetration has been proposed in the formation of such mesiodens. In twins, unilateral mesiodens may present as mirror images located in similar regions of the mouth in the same number [5].

Types of mesiodens

Mesiodens teeth can be classified based on their occurrence in the permanent dentition (rudimentary mesiodens), which are usually smaller and abnormal in shape, or the primary dentition (supplementary mesiodens), which resemble natural teeth in both size and shape [6]. Based on the morphology (conical, tuberculate, or molariform), conical mesiodens usually occur singly. They are generally peg-shaped and usually located palatally between the maxillary central incisors, tending to displace the erupting permanent central incisors [6-8].

Conical mesiodens often can erupt into the oral cavity and have a completely formed root [9]. The crown can be inverted pointing superiorly, in which case they are less likely to erupt into the oral cavity; inverted conical mesiodens have occasionally erupted into the nasal cavity [8]. Tuberculate mesiodens teeth are barrel-shaped, with several tubercles or cusps, and have incomplete or abnormal root formation. In contrast to conical mesiodens, tuberculate mesiodens teeth rarely erupt themselves but rather develop either unilaterally or bilaterally and delay the eruption of the permanent incisors [8]. Tuberculate mesiodens teeth develop later than conical mesiodens and usually occupy a more palatal position [8]. A third, much rarer type is the molariform mesiodens, which has a crown resembling a premolar tooth and a completely formed root. Various research studies on mesiodens by CBCT are enumerated in Table 1.

**Table 1:** Research studies on mesiodens by CBCT CBCT: cone-beam computed tomography.

Author	Year	Age/gender	Shape/position/number of mesiodens	Clinical description of mesiodens
Kim et al. [8]	2013	Radiographic evaluation of 280 pediatric patients	Inverted	The majority of the mesiodens were conical (79.5%) in shape and inverted (48.6%) in direction. Inverted mesiodens teeth were present among 235 out of 280 (61%) patients. CBCT is an excellent diagnostic tool, providing three-dimensional information on impacted mesiodens
Omami et al. [7]	2017	8-year-old/female	Inverted and palatally placed	CBCT yielded accurate three-dimensional information of supernumerary tooth mesiodens relative to the orientation, sagittal position, local disorders, and neighboring anatomic structures and hence has great significance for pretreatment evaluation of supernumeraries
Kim et al. [10]	2017	293 Korean children/4-10 years	Inverted and palatally placed	Inverted mesiodens occurred in 228 out of 298 (59.5%) patients. Mesiodens caused a delay in maxillary incisor eruption-related complications in 33.7% of patients
Goksel et al. [4]	2018	Retrospective study of 5,000 CBCT scans collected from December 2015 to March 2018	Bilateral	In 19 out of 101 cases, mesiodens teeth were bilateral (18.8%). CBCT provides more detailed information about the position, neighboring anatomic structures, and local findings of the presence of mesiodentes in multiplanar sections
Beschiu et al. [5]	2021	Case study of an ancient skeleton collected from an archaeological site in the western part of Romania	Mesiodens vertical in line with mid palatal suture	Mesiodens is an anomaly found throughout all historical periods, from the oldest archaeological sites to the present day. The maximal crown root distance of mesiodens from the cementoenamel junction to the incisal margin is 8.99 mm. Mesiodistal diameter is 5.05 mm
Perez et al. [11]	2022	13-year-old patient	Inverted, class 4 mesiodens (angulation between 90 and 180 degrees)	The tortuosity of mesiodens was discovered by CBCT

Mupparapu et al.'s classification of mesiodens is mentioned in Table 2 [12].

**Table 2:** Mupparapu's classification of mesiodens Adapted from [12].

Class of mesiodens	Description of mesiodens
Class 1	Impacted mesiodens is parallel or 0 degrees to the normal eruptive pattern of maxillary central incisors
Class 2	Impacted mesiodens is between 0 and 90 degrees from the normal eruptive pattern
Class 3	Impacted mesiodens is perpendicular or 90 degrees to the normal eruptive pattern
Class 4	Impacted mesiodens is between 90 and 180 degrees from the normal eruptive pattern
Class 5	Impacted mesiodens is inverted or 180 degrees to the normal eruptive pattern

Mesiodens can be removed by a conservative surgical approach by frenulum incision and lateral tunneling and packing the surgical defect with platelet-rich plasma fibrin, minimizing scar and thereby improving the aesthetic outcome [12].

## Conclusions

The most common supernumerary tooth that usually occurs in the maxillary anterior region is the mesiodens between the two maxillary central incisors. Mesiodens is also termed peridens (near the premolar) and distodens (near the molar). Mesiodens can lead to midline diastema and recurrent rhinitis. A CBCT is essential for properly evaluating its location in a three-dimensional view for its treatment planning. Before surgical removal of symptomatic mesiodens, a labial or palatal approach to mesiodens can be planned after radiographic evaluation by CBCT.
